# Macrophage Migration Inhibitory Factor and Malondialdehyde as Potential Predictors of Vascular Risk Complications in Type 2 Diabetes Mellitus: Cross-Sectional Case Control Study in Saudi Arabia

**DOI:** 10.1155/2016/5797930

**Published:** 2016-05-19

**Authors:** Heba Kamal Morsi, Manar Mohammad Ismail, Hassan Abdelaziz Hassan Gaber, Amani Abdelhamid Elbasmy

**Affiliations:** ^1^Medical Biochemistry Department, Faculty of Medicine, Mansoura University, Mansoura 35516, Egypt; ^2^Laboratory Medicine Department, College of Applied Medical Science, Umm Al Qura University, Makkah 21955, Saudi Arabia; ^3^Clinical Pathology Department, National Cancer Institute, Cairo University, Cairo 11796, Egypt; ^4^Clinical Pathology Department, Al Azhar University, Cairo 11651, Egypt; ^5^Clinical Chemistry Department, Al Noor Specialist Hospital, Makkah 21955, Saudi Arabia; ^6^Cancer Epidemiology and Biostatistics Department, National Cancer Institute, Cairo University, Cairo 11796, Egypt; ^7^Kuwait Cancer Registry, 70653 Kuwait, Kuwait

## Abstract

*Background*. Malondialdehyde (MDA) has been implicated in the development of many acute inflammatory, autoimmune diseases as well as chronic inflammatory metabolic disorders. Involvement of inflammatory response and oxidative stress is currently suggested as a mechanism underlying development of diabetes and its complications.* Objective*. To evaluate the clinical utility of MDA, macrophage migration inhibitory factor (MIF), LDL-C/HDL-C, and TG/HDL-C ratio as noninvasive laboratory markers for prediction of T2DM vascular complications.* Method*. 63 Saudi T2DM patients and 16 age and sex matched controls were included. Serum MDA and MIF were assayed by thiobarbituric acid reactive substances and ELISA, respectively. TG/HDL-C and LDL-C/HDL-C ratios were calculated.* Results*. Uncontrolled DM patients had significantly higher levels of MDA, MIF, TG/HDL-C, and LDL-C/HDL-C ratios when compared with controlled DM patients and control group (*p* < 0.001). MDA had 100% sensitivity and 88% specificity. MIF showed 97% sensitivity and 100% specificity and LDL-C/HDL-C had 97% sensitivity and 95% specificity. Meanwhile, TG/HDL-C had the lowest sensitivity and specificity in identifying diabetic patients who would suffer from vascular complications.* Conclusion*. MDA, MIF, and LDL-C/HDL-C could be new predictors of metabolic disturbance which promote vascular complications in T2DM.

## 1. Introduction

Diabetes mellitus (DM) is one of the most frequent chronic metabolic diseases worldwide, being among the top five main causes of death in developed countries. Also, it is becoming an epidemic in developing countries [1]. In 2013, International Diabetes Federation (IDF) estimated that 382 million people have diabetes which is expected to rise to 592 million by 2035 [2].

Type 2 diabetes mellitus (T2DM) accounts for ~90–95% of all diabetes cases. The two metabolic defects that characterize T2DM are a decrease response of peripheral tissue to insulin (insulin resistance) and failure of insulin secretion by pancreatic *β*-cells. The most common risk factors for this type of diabetes are genetic conditions, obesity, lifestyle, and eating habits [3]. Saudi Arabia is among the world's leaders in terms of T2DM prevalence. Based on the Ministry of Health and Ministry of Finance database, 0.9 million people in 1992 and 2.5 million people in 2010 have been diagnosed with diabetes. This increased prevalence of diabetes is attributable not only to changing pattern of Saudi lifestyle due to rapid socioeconomic development but also to increased awareness programs related to diabetes and its health complications, community screening campaigns, better diagnostic facilities, and better diabetes management systems and protocols [4].

DM is characterized by chronic hyperglycemia with disturbances of carbohydrate, fat, and protein metabolism that mostly ends in damage to and/or malfunction of various organs, including high risk of cardiovascular diseases (CVD) [3]. In T2DM, the high risk of CVD is due to an accelerated atherosclerotic process resulting from disruption of insulin regulatory role in lipid and lipoprotein metabolism. One important cardiovascular risk is dyslipidemia that was defined by National Cholesterol Education Program Adult Treatment Panel III (NCEP-ATP III) guideline by presence of one or more of the following: hypercholesterolemia as total cholesterol (TC) more than 200 mg/dL, high level of low-density lipoprotein cholesterol (LDL-C) that is more than 100 mg/dL, hypertriglyceridemia as triglycerides (TG) more than 150 mg/dL, and low level of high-density lipoprotein cholesterol (HDL-C) as less than 40 mg/dL [5]. However, LDL-C/HDL-C ratio is considered by some authors as a better atherogenic index than LDL-C that measures the “bad” cholesterol or HDL-C which is a measure of “good” cholesterol or TC which is the sum of HDL, LDL, and VLDL [6] and calculation of TG/HDL-C ratio may be used as a tool to evaluate the efficiency with which a lipid load is removed from the circulation [7].

A serious imbalance between reactive species production and antioxidant defenses is referred to as oxidative stress (OS) and usually associated with oxidative damage [8]. Hydroxyl radicals initiate a free radical chain reaction and remove hydrogen atom from one of the carbon atoms in the polyunsaturated fatty acids in the plasma membrane and lipoproteins causing lipid peroxidation. Lipid peroxides decompose to form toxic and reactive compounds such as MDA, a stable end product that can serve as a reliable marker for the assessment of free radical induced damage to tissues [9]. Oxidative damage to the cells ultimately results in secondary complications of DM [10].

Macrophage migration inhibitory factor (MIF) is a cytokine that is expressed both by immune and by nonimmune cells. It is well known for its proinflammatory effects [11] through its enzymatic tautomerase and oxidoreductase activity [12] and it can recruit inflammatory cells via chemokine signaling [13]. So, it has been implicated in the development of many acute inflammatory and autoimmune diseases [14] as well as chronic inflammatory metabolic disorders [15]. MIF colocalizes in secretory insulin granules within *β*-cells and it is released during insulin secretion, suggesting an autocrine, glucose-dependent regulatory effect on insulin secretion [16]. Inflammation has been proposed to contribute to *β*-cell dysfunction in diabetes [17, 18]. Indeed, islets from diabetic patients show immune cell infiltration and increased cytokine and chemokine expression [19]. Therefore, involvement of the inflammatory response is equally important in disease development and complications, hence the reason why the role of MIF has been studied in T2DM [20].

*Objective*. To evaluate the clinical utility of MDA, MIF, and lipid risk factors LDL-C/HDL-C and TG/HDL-C ratio as noninvasive laboratory markers for prediction of T2DM vascular complications, especially in patient with poor glycemic control.

## 2. Subjects and Methods

### 2.1. Subjects

Sixty-three T2DM patients of both sexes and sixteen healthy volunteers with comparable age and sex as control were included in this study. The patients were selected from those regularly attending the Diabetes Clinic in Al Noor Specialist Hospital, Makkah, from June 2013 to August 2013. Full medical history was taken and clinical examination was performed. The patients were diagnosed as type II diabetics according to the World Health Organization Consultation and International Expert Committee [21, 22].

#### 2.1.1. Exclusion Criteria

T2DM patients who complain of cardiovascular, liver, and kidney diseases, other endocrine disorders, and acute or chronic inflammatory diseases as well as those who were on antioxidants supplementation in the previous two months were excluded from the study. Also Type 1 DM patients were excluded from the study.

On the basis of blood hemoglobin A1c (HbA1c)%, indicator of glycemic control, T2DM patients were categorized into two groups.

*Controlled DM*: 27 patients with HbA1c level ≤ 7% reflecting good glycemic control. They were 16 male and 11 female patients. Their age range was 39–65 and mean ± SD was 53.03 ± 7.2 years.

*Uncontrolled DM*: 36 patients with HbA1c level > 7% reflecting poor glycaemic control. They were 18 male and 18 female patients. Their age range was 40–65 and mean ± SD was 53.7 ± 5.9 years.

The study was performed in accordance with the principles of Declaration of Helsinki and approved by the ethics committee of Faculty of Applied Medical Sciences, Umm Al Qura University, Saudi Arabia, and written informed consent was obtained from all participants involved in the study.

### 2.2. Samples

Five milliliters of venous blood was withdrawn from all participants after 12 hours of fasting and before taking medications. The blood samples were collected into EDTA vacutainers for HbA1c assay and serum separator tubes. Serum was separated by centrifugation for 15 minutes at 1500 ×g for assay of routine laboratory tests. The rest of the serum was divided into aliquots and stored at −20°C until analysis of MDA and MIF.

### 2.3. Methods

MDA (R&D Systems, Inc., USA) was measured spectrophotometrically by thiobarbituric acid reactive substances [23] according to the manufacturer's instructions. MIF (R&D Systems, Inc., USA) was assayed by quantitative sandwich enzyme linked immune assays according to the manufacturer's instructions. Routine tests include fasting blood glucose (FBG), lipid profile; TG, TC, LDL-C, and HDL-C, kidney functions tests; serum creatinine and urea, liver functions tests; serum activity of alanine amino transferase (ALT), aspartate amino transferase (AST), alkaline phosphatase (ALP), total bilirubin level, and HbA1c% were analyzed using Dimension EXL analyzer (Siemens, Germany). LDL-C/HDL-C and TG/HDL-C ratios were calculated.

### 2.4. Statistical Methods

Data were analyzed using SPSS (Statistical Package for Social Sciences; SPSS Inc., Chicago, IL, USA) version 20 for Microsoft Windows. Numerical data were presented as mean ± SD, median, and range as appropriate. Comparisons between different groups of numerical data were conducted using ANOVA (analysis of variance) and paired comparisons were conducted using Bonferroni's test. Pearson's correlation was used for correlation analysis. Probability (*p* value) < 0.05 was considered significant and highly significant if *p* values were <0.001. Receiver operating characteristic (ROC) curve was conducted for the potential studied risk factors to calculate sensitivity and specificity.

## 3. Results

Basic laboratory investigations were presented in Table 1.

### 3.1. Fasting Blood Glucose Level and HbA1c% among the Different Studied Groups

Uncontrolled DM group showed highly significant increase of FBG level and HbA1c% compared to both control and controlled DM groups (*p* value = 0.001). The controlled DM group did not differ significantly from the control group in FBG level (*p* value = 0.11), while it showed a significantly higher HbA1c% than the control group (*p* value = 0.02).

### 3.2. Lipid Profile among the Different Studied Groups

There was a highly significant increase in TC level in both uncontrolled and controlled DM patients when compared with the control group. Also, the uncontrolled DM patients showed highly significant increase from the controlled DM patients (*p* value < 0.001). Both uncontrolled and controlled DM groups had a highly significant lower level of HDL-C when compared with the control group. Also, there was highly significant decrease of HDL-C level in uncontrolled DM group when compared with controlled group (*p* value = 0.001). The uncontrolled DM group had highly significant increase of TG and LDL-C compared with normal control and controlled DM groups (*p* value = 0.001), while no significant difference was observed between controlled DM and normal control (*p* value = 0.05).

### 3.3. MIF Level among the Different Studied Groups

MIF levels were significantly elevated in uncontrolled DM group compared with both control and controlled DM groups (*p* value < 0.001). Also, it was significantly elevated in controlled DM in comparison with control group (*p* value = 0.03).

### 3.4. MDA Level among the Different Studied Groups

MDA levels in the uncontrolled DM group showed highly significant increase when compared with both control and controlled DM groups (*p* value < 0.001), while there was no statistically significant difference between the controlled DM and control group (*p* value > 0.05).

### 3.5. LDL-C/HDL-C and TG/HDL-C Ratios

The uncontrolled DM group had highly significant elevated LDL-C/HDL-C and TG/HDL-C ratios compared to normal control and controlled DM groups (*p* value < 0.001), while controlled DM group did not significantly differ from normal control (*p* value > 0.05). Data were presented in Table 2.

**Figure 1:**
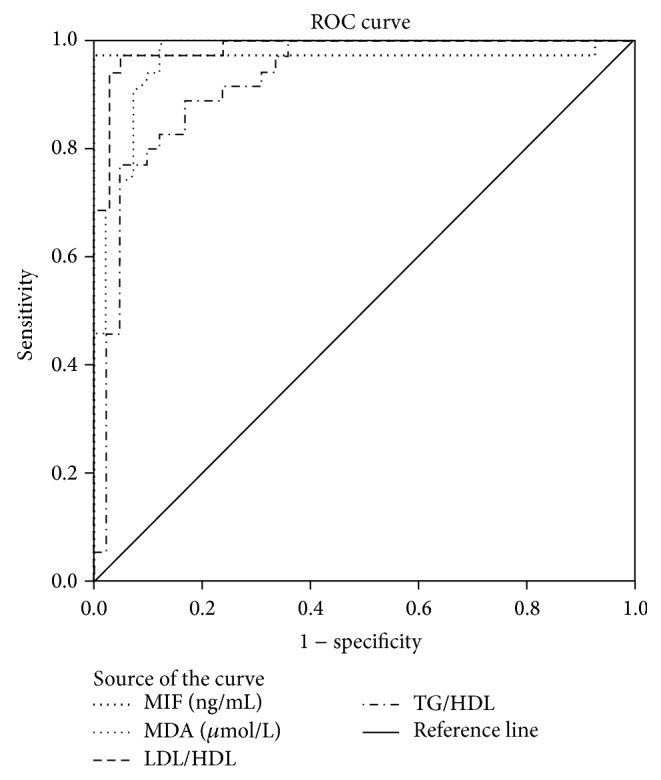
ROC curve analysis of the studied potential predictors of diabetic complications.

Correlations between the serum levels of each MDA, MIF, LDL-C/HDL-C, and TG/HDL-C ratios and FBG and HbA1c as well as lipid profile were presented in Table 3. Receiver operating characteristic (ROC) curve was plotted for the uncontrolled DM group versus the rest of the studied cases to calculate the best cutoff value, sensitivity, and specificity of MIF and MDA levels and TG/HDL-C and LDL-C/HDL-C ratios in identifying diabetic patients who would suffer from the complications. Accuracy was measured by the area under the ROC curve (AUC). AUC for all the parameters is more than 0.9, meaning that they are excellent tests for prediction of diabetic complications. The data is presented in Table 4 and Figure 1.

## 4. Discussion

Oxidative stress (OS) results when there is increased production of free radicals or decreased activity of counter-actors, antioxidants, or both in a combination. OS plays a pivotal role in progression and development of diabetes and its complications [24].

Both diabetic groups in the current study exhibited highly significant increase of TC and highly significant decrease of HDL-C compared with the control group. However, only uncontrolled diabetics had significantly higher TG, LDL-C, TG/HDL-C, and LDL-C/HDL-C ratio when compared with the control and controlled DM groups, in agreement with Bonfanti et al. [25]. These findings indicate dyslipidemia according to NCEP-ATP III [5] and could be attributed to OS [26], decreased activity of lipoprotein lipase and cholesterol ester transport protein [27], and increased free fatty acid mobilization from the liver [28].

Hypertriglyceridemia leads to an increased production of a more atherogenic, small, dense form of LDL-C and decreased cholesterol transport to the liver by HDL. Therefore, high value of TG/HDL-C ratio in present work can enable identification of diabetic patients at a higher risk of CVD. Moreover, it could be a contributing factor to pancreatic lipotoxicity, *β*-cell failure, and poor glycemic control as reported by Ebesunun and Adedipe [29].

In agreement with former studies, we found higher serum MIF levels in diabetic groups than in healthy individuals and in addition it was higher in uncontrolled than in controlled DM groups [30, 31]. Under physiological conditions, MIF is produced by pancreatic *β*-cells and maintains insulin secretion activity [32, 33], while in altered homeostasis, MIF acts as a booster of inflammation that underlines the development of T2DM [34] MIF can exacerbate insulitis, local pancreatic inflammation, and contribute to *β*-cells apoptosis [35] and finally *β*-cells dysfunction [36]. It is estimated that up to 25% of newly diagnosed T2DM patients already had evidence of systemic inflammation at the time of diagnosis [37]. In the same context, MIF stimulates the inflammatory adipocytokines; resistin and IL-6 both are key molecules in the development of insulin resistance [38].

Moreover, MIF is a necessary mediator of TNF-*α*, which inhibits the insulin signal transduction leading to insulin resistance. It inhibits phosphorylation of the protein kinase AKT that together with a phosphatidylinositol 3-kinase is necessary for phosphorylation of insulin receptor substrate-1 which stimulates transcription of insulin-regulated genes [39]. These could explain the highly significant positive correlations found in this study between MIF, FBG, and HbA1c%. This is in agreement with the study done by Yu et al. [40] proving the role of MIF in the development of T2DM.

High MIF levels act as a recruitment factor for inflammatory cells to one of our vital organs, the liver. These inflammatory cells can mediate hepatocellular injury [41]. However, OS and insulin resistance could be a contributing factor to liver damage [42]. These could explain high ALP activity in uncontrolled DM group in this study.

MIF was positively correlated with MDA which is in consistence with previous studies [43, 44]. This reflects the possible role of OS in induction of MIF release [45]. However, positive correlation of MIF with TG/HDL-C ratio might reflect an induction of MIF by high FFA which characterizes the insulin resistance and T2DM [46]. This may explain the high level of MIF in uncontrolled cases carrying higher incidence of complications.

In the present study, uncontrolled diabetics had significantly higher levels of MDA, an indicator of lipid peroxidation in agreement with Bikkad et al. and Shinde et al. [47, 48]. In agreement with Manohar et al., we found significant positive correlation between MDA level and all measured lipid parameters except HDL-C that was negatively correlated with MDA, denoting the coexistence of atherogenic risk factors and OS [49]. Furthermore, MDA levels show significant positive correlations with indicators of glycemic control, FBG and HbA1c%. One of the derangement effects of OS in diabetics is disturbance of renal functions [50]; that may explain the significant higher blood urea level detected in the uncontrolled than in the controlled diabetic group.

High MDA level may be the end result of chronic hyperglycemia effect in the development of OS. It is well known that chronic hyperglycemia due to either decreased insulin secretion or insulin resistance leads to excess free radical generation with a consequent lipid peroxidation, depletion of antioxidants, and enhanced OS in T2DM [51]. Hyperglycemia causes the production of free radicals in several ways: firstly, through nonenzymatic generation of advanced glycation end products which are highly reactive, causing cross link formation, trapping of proteins, and lipid peroxidation [52]. Secondly, hyperglycemia generates greater amounts of superoxide anions more than the scavenging capacity of mitochondrial superoxide dismutase [53]. Thirdly, hyperglycemia decreases intracellular NADPH content, resulting in depletion of reduced glutathione [54]. Those together with decreased activity of the antioxidant enzymes by glycation as well as their consumption lead to an increased OS [55]. However, OS produces insulin resistance via inactivation of glucose transporter 4 with subsequent hyperglycemia. Eventually, a vicious circle links hyperglycemia to OS and establishment of micro- and macrovascular complications in T2DM [56].

Based on ROC curve analysis, we found that MIF and MDA had higher sensitivity and specificity than lipid risk factors TG/HDL-C and LDL-C/HDL-C ratio. Although the sensitivity of MDA was higher than that of MIF (100% versus 97%), the specificity of MIF was higher than that of MDA (100% versus 88%). Out of the studied markers, TG/HDL-C ratio had the lowest sensitivity and specificity in identifying diabetic patients at higher risk of developing complications. Thus, high sensitivity and specificity of MIF, MDA, and LDL-C/HDL-C indicate the importance of these parameters as predictors of metabolic disturbances which promote diabetic vascular complications.

## 5. Conclusion

MIF, MDA, and LDL-C/HDL-C could be predictors of metabolic disturbances which promote vascular complications in T2DM patients and might provide an early sensitive screening tool for optimal management of T2DM patients. Also, targeting MIF by a novel therapy together with antioxidant may guard against the development of diabetic complications. Measurement of MIF and MDA level and calculation of LDL-C/HDL-C in T2DM patients are strongly recommended.

## Figures and Tables

**Table 1:** Basic laboratory investigations in the different studied groups.

Variables	Normal control *n* = 16	Controlled DM *n* = 27	Uncontrolled DM *n* = 36	*p* value
FBG (mg/dL)				
Mean ± SD	85.19 ± 7.2	116.33 ± 18.08	192.5 ± 66.8	<0.001
Median (min–max)	84.5 (77–100)	122 (83–142)	175 (99–373)^b*∗∗*&c*∗∗*^	
HbA1c (%)				
Mean ± SD	4.91 ± 0.44	5.9 ± 0.83	9.65 ± 1.48	<0.001
Median (min–max)	4.95 (4–5.5)	6 (4–7.5)^a*∗*^	9.75 (7.3–12.4)^b*∗∗*&c*∗∗*^	
Albumin (g/dL)				
Mean ± SD	4.18 ± 0.31	3.2 ± 0.8	3.5 ± 0.72	<0.001
Median (min–max)	4.1 (3.8–4.8)	3.1 (2–4.8)^a*∗∗*^	3.7 (1.6–4.6)^b*∗*^	
Total bilirubin (mg/dL)				
Mean ± SD	0.61 ± 0.25	0.56 ± 0.39	0.9 ± 1.43	0.35
Median (min–max)	0.54 (0.2–1)	0.4 (0.1–1.6)	0.5 (0.1–6.7)	
Total protein (g/dL)				
Mean ± SD	7.17 ± 0.71	6.73 ± 1.18	7.03 ± 1.33	0.45
Median (min–max)	7.15 (5.9–8.3)	6.5 (3.7–8.9)	7.15 (1–10)	
ALT (U/L)				
Mean ± SD	16.75 ± 3.55	42.19 ± 27.4	38.47 ± 30.2	0.15
Median (min–max)	17 (11–21)	31 (8–95)	28.5 (10–172)	
AST (U/L)				
Mean ± SD	23.56 ± 5.30	46.1 ± 50.16	30.81 ± 39.13	0.14
Median (min–max)	23 (12–31)	29 (10–211)	19.5 (10–241)	
ALP (U/L)				
Mean ± SD	49.88 ± 5.01	90.93 ± 47.98	124.3 ± 96.76	0.003
Median (min–max)	50 (40–57)	78 (23–222)	103.5 (18–623)^b*∗*^	
Urea (mg/dL)				
Mean ± SD	21.75 ± 3.1	33.93 ± 28.2	44.88 ± 34.93	0.03
Median (min–max)	21 (17–28)	26.5 (6–152)	33 (0.8–161)^b*∗*^	
Creatinine (mg/dL)				
Mean ± SD	0.83 ± 19	1.3 ± 1.22	1.24 ± 1.53	0.45
Median (min–max)	0.8 (0.6–1.2)	0.8 (0.4–4.5)	0.8 (0.4–8.9)	
TC (mg/dL)				
Mean ± SD	62.31 ± 7.6	105.67 ± 34.27	182.89 ± 40.18	<0.001
Median (min–max)	60.5 (50–74)	100 (60–220)^a*∗∗*^	186.5 (101–266)^b*∗∗*&c*∗∗*^	
TG (mg/dL)				
Mean ± SD	52.73 ± 7.16	103.41 ± 56.09	183.11 ± 101.96	<0.001
Median (min–max)	52.5 (41–67)	88 (50–339)	177 (78–505)^b*∗∗*^	
LDL-C (mg/dL)				
Mean ± SD	63.63 ± 5.86	64.85 ± 20.25	134.41 ± 36.9	<0.001
Median (min–max)	62 (56–75)	70 (6–90)	134.5 (70–212)^b*∗∗*&c*∗∗*^	
HDL-C (mg/dL)				
Mean ± SD	63.06 ± 11.62	48.92 ± 12.2	35.89 ± 9.7	<0.001
Median (min–max)	64 (35–80)	49 (28–90)^a*∗∗*^	35 (7–64)^b*∗∗*&c*∗∗*^	

^a^Controlled DM versus control group.

^b^Uncontrolled DM versus control group.

^c^Uncontrolled DM versus controlled DM groups.

*n*: number of cases.

^*∗*^Significant (*p* value < 0.05).

^*∗∗*^Highly significant (*p* value < 0.001).

**Table 2:** Levels of the studied potential predictors of diabetic complications in the different group.

Parameters	Normal control *n* = 16	Controlled DM *n* = 27	Uncontrolled DM *n* = 36	*p* value
MIF (ng/mL)				
Mean ± SD	1.8 ± 0.38	3.79 ± 0.75	9.58 ± 1.66	<0.001
Median (min–max)	1.8 (1.09–2.5)	4.07 (0.9–4.6)^a*∗*^	9.7 (1.1–11.2)^b*∗∗*&c*∗∗*^	
MDA (nmol/L)				
Mean ± SD	1.23 ± 0.67	1.5 ± 0.95	4.5 ± 1.5	<0.001
Median (min–max)	1.43 (0.09–2.1)	1.014 (0.58–4.05)	3.88 (2.2–7.75)^b*∗∗*&c*∗∗*^	
LDL-C/HDL-C				
Mean ± SD	0.99 ± 0.20	1.35 ± 0.59	4.41 ± 4.05	<0.001
Median (min–max)	1.04 (0.48–1.22)	1.25 (0.0–3.04)	3.64 (1.52–27)^b*∗∗*&c*∗∗*^	
TG/HDL-C				
Mean ± SD	0.869 ± 0.236	2.36 ± 2.11	5.47 ± 3.24	<0.001
Median (min–max)	0.79 (0.58–1.51)	1.88 (0.89–12.11)	4.62 (1.7–14.43)^b*∗∗*&c*∗∗*^	

^a^Controlled DM versus control group.

^b^Uncontrolled DM versus control group.

^c^Uncontrolled DM versus controlled DM groups.

*n*: number of cases.

^*∗*^Significant (*p* value < 0.05).

^*∗∗*^Highly significant (*p* value < 0.001).

**Table 3:** Pearson's correlation between potential predictors of diabetic complications and basic laboratory investigations.

Parameters	MIF (ng/mL)		MDA (*μ*mol/L)		LDL-C/HDL-C		TG/HDL-C	
	*R*	*p*	*R*	*p*	*R*	*p*	*R*	*p*
FBG (mg/dL)	0.26	0.02	0.69	<0.001	0.26	0.02	0.38	0.001
HbA1c (%)	0.35	0.001	0.81	<0.001	0.37	0.001	0.54	<0.001
TC (mg/dL)	0.4	<0.001	0.72	<0.001	0.32	0.004	0.55	<0.001
TG (mg/dL)	0.16	0.17	0.35	0.002	0.197	0.08	0.88	<0.001
HDL-C (mg/dL)	−0.32	0.004	−0.4	<0.001	−0.57	<0.001	−0.64	<0.001
LDL-C (mg/dL)	0.51	<0.001	0.65	<0.001	0.64	<0.001	0.51	<0.001
TG/HDL-C	0.29	<0.01	0.33	0.003	0.58	<0.001	—	—
LDL-C/HDL-C	0.29	0.01	0.3	0.007	—	—	0.58	<0.001
MDA (*μ*mol/L)	0.4	<0.001	—	—	0.3	0.007	0.33	0.003
MIF (ng/mL)	—	—	0.4	<0.001	0.29	0.01	0.29	<0.01

*R* value: ≥0.7 = strong linear relationship, ≥0.5 = moderate linear relationship, and ≥0.3 = weak linear relationship.

Negative value = downhill relationship, positive value = uphill relationship.

**Table 4:** Diagnostic accuracy of potential predictors of diabetic complications.

Markers	AUC	Cutoff	Sensitivity	Specificity	PPV	NPV
MIF	0.97	6.55	97%	100%	97%	100%
MDA	0.97	2.2	100%	88%	87%	100%
LDL-C/HDL-C	0.98	1.96	97%	95%	94%	97%
TG/HDL-C	0.92	2.22	89%	81%	80%	89%
